# Effect of low-frequency repetitive transcranial magnetic stimulation combined with abdominal acupuncture on lower limb motor function and balance in post-stroke hemiplegia: a randomized controlled trial

**DOI:** 10.3389/fneur.2026.1870613

**Published:** 2026-08-04

**Authors:** Chaobing Zhang, Shixin Qin, Xiuyuan Wang, Li Shu, Xueping Dong

**Affiliations:** Department of Rehabilitation Medicine, The Third People’s Hospital of Hefei, Hefei Third Clinical College of Anhui Medical University, Hefei, China

**Keywords:** abdominal acupuncture, hemiplegia, lower limb dysfunction, repetitive transcranial magnetic stimulation, stroke

## Abstract

**Background:**

Post-stroke hemiplegia frequently results in lower limb motor dysfunction and impaired balance, leading to reduced independence in activities of daily living. Repetitive transcranial magnetic stimulation (rTMS) and abdominal acupuncture (AA) are both used in rehabilitation, but evidence on their combined effect remains limited. This study evaluated the efficacy and safety of low-frequency rTMS combined with AA in patients with post-stroke hemiplegia.

**Methods:**

In this assessor-blind randomized controlled trial, 108 patients with post-stroke hemiplegia and lower limb dysfunction were randomly assigned (1:1:1) to a control group (conventional rehabilitation), an AA group (rehabilitation plus abdominal acupuncture), or a combined group (rehabilitation plus AA and low-frequency rTMS). All interventions were administered for 8 weeks. Outcomes were assessed at baseline, 4 weeks, and 8 weeks. The primary outcomes included lower limb motor function measured by the Fugl-Meyer Assessment of Lower Extremity (FMA-LE), balance assessed by the Berg Balance Scale (BBS), and activities of daily living evaluated using the Modified Barthel Index (MBI). Adverse events were recorded to assess safety.

**Results:**

All groups showed significant improvement in FMA-LE, BBS, and MBI scores at 4 and 8 weeks compared with baseline (all *p* < 0.05). The combined group demonstrated significantly greater improvements than both the AA and control groups at each post-treatment time point (*p* < 0.05), while the AA group also showed superior outcomes compared with the control group (*p* < 0.05). At 8 weeks, the combined group achieved the highest scores across all outcomes. No serious adverse events were recorded during the trial, while several mild transient adverse events were observed in a small proportion of participants. And the incidence of mild adverse events did not differ significantly among groups (*p* > 0.05).

**Conclusion:**

Low-frequency rTMS combined with abdominal acupuncture significantly improves lower limb motor function, balance, and activities of daily living in patients with post-stroke hemiplegia, with a favorable safety profile. This combined approach may offer an effective strategy for enhancing rehabilitation outcomes.

**Clinical trial registration:**

https://itmctr.ccebtcm.org.cn, identifer ITMCTR2026001195.

## Introduction

Stroke is characterized by a high incidence rate, recurrence, disability, and mortality, making it a significant concern for public health worldwide (1). Although advancements in medical technology have reduced the fatality rate, many survivors continue to face severe long-term consequences. Approximately 70% of stroke survivors experience motor dysfunction, particularly affecting the lower limbs (2). This dysfunction can profoundly impact balance, mobility, and activities of daily living (ADL), placing a substantial burden on both patients and their families. Post-stroke hemiplegia and lower limb dysfunction primarily stem from neuronal damage and the disruption of neural pathways (3). Local brain tissue suffers from insufficient blood supply due to either blockage or rupture of blood vessels, which leads to neuronal apoptosis and the activation of glial cells. This form of damage affects not only the core area of the lesion but also other brain regions that are functionally connected, resulting in lower limb dysfunction (4). Furthermore, the disruption of neural conduction pathways, particularly the damage to the corticospinal tract, is a significant contributor to abnormal activation patterns in lower limb muscles, reduced muscle strength, and diminished coordination (5).

The current landscape of clinical rehabilitation treatments, namely physical therapy, occupational therapy, and traditional Chinese medical practices, reveals significant limitations in enhancing lower limb function (6). Physical therapy often requires a long-term commitment, and its effectiveness can vary considerably among individuals due to factors such as training intensity and adherence (7). While occupational therapy focuses on improving ADL, it does not adequately address lower limb motor function. Additionally, traditional Chinese medical therapies, including acupuncture and massage, typically utilize a singular treatment approach, thereby missing the potential benefits of integrating multiple methods. These challenges highlight the urgent need for more targeted and integrative treatment plans tailored specifically for patients in the plateau stage of functional recovery (Brunnstrom stages II-IV) (8). This stage represents not just a transition but a crucial opportunity for remodeling motor functions, making it essential to prioritize the development of comprehensive strategies that maximize outcomes during this vital period.

Repetitive transcranial magnetic stimulation (rTMS) is a non-invasive technology designed for brain neural regulation, recognized for its simplicity and high safety profile (9). It allows for personalized modulation of the excitability in either the affected or healthy hemisphere of the brain, and substantial evidence supports its efficacy in rehabilitating limb motor function following a stroke (10). Neuroscience research has demonstrated that a mediated inhibitory balance exists between the two cerebral hemispheres via the corpus callosum. After a stroke, pathological changes such as hemorrhage or infarction can impair the inhibitory effect that the affected hemisphere exerts on the healthy hemisphere (11). This leads to an imbalance characterized by reduced excitability in the affected hemisphere and an abnormal increase in excitability in the healthy hemisphere. Low-frequency rTMS (⩽1 Hz) specifically targets the excessive excitation in the healthy hemisphere, facilitating the restoration of a dynamic balance in excitability between both sides of the brain (12). The primary mechanism of rTMS is ‘local regulation of the central nervous system’, which focuses on addressing the underlying pathologies affecting the central nervous system.

Abdominal acupuncture (AA) therapy is a distinctive technique based on traditional Chinese medicine, increasingly recognized for its painlessness, safety, and effectiveness in post-stroke rehabilitation (13). Developed by Professor Zhiyun Bao, this therapy utilizes the entire abdomen to identify acupuncture points, facilitating the regulation of qi and blood circulation within internal organs and ensuring a harmonious meridian system. Recent studies have demonstrated that AA can stimulate acupuncture points associated with abdominal meridians and internal organs to activate the neural-endocrine-immune regulatory mechanisms, promoting structural remodeling and functional recovery of damaged nerve tissues (14). AA emphasizes ‘holistic regulation of the peripheral circulatory system’, employing techniques such as enhancing blood flow, adjusting qi, and relaxing meridians to target specific areas. Ultimately, the goal is to establish a stable state of balance and harmonize the functions of internal organs.

In recent years, the use of rTMS and AA in stroke rehabilitation has grown significantly. However, most existing studies focus solely on one technique or treatment for upper limb dysfunction (15, 16). Recent meta-analysis results indicated that the combined treatment approach of acupuncture combined with rTMS has superior efficacy in improving the upper limb motor function of stroke patients, enhancing their daily living abilities, and repairing nerve damage compared to using acupuncture or rTMS alone (17). The positive efficacy evidence from upper limb-focused studies provides a solid theoretical and clinical foundation for further exploring the value of combination therapy in addressing lower limb dysfunction. Therefore, this study aims to evaluate the effectiveness of rTMS combined with AA in improving lower limb motor function, balance ability, and ADL in patients experiencing lower limb dysfunction due to post-stroke hemiplegia.

## Methods

### Study design

This study was conducted as an assessor-blind randomized controlled trial, receiving approval from the Ethics Committee of Hefei Third People’s Hospital (2024LLW073). All patients involved in the trial were thoroughly informed of the study details and signed the informed consent form.

### Participants

Patients with post-stroke hemiplegia and lower limb dysfunction from January 2024 to December 2025 meeting the following criteria were included in this study: (1) a confirmed diagnosis of hemorrhagic or ischemic stroke, validated through cranial CT or MRI imaging; (2) first occurrence of stroke with a disease duration of less than 3 months; (3) normal cognitive function to facilitate cooperation during treatment and assessment; (4) unilateral lower limb motor dysfunction classified within Brunnstrom stages II to IV.

The exclusion criteria were: (1) serious medical conditions or concurrent significant organ dysfunction, including issues with the heart, lungs, liver, or kidneys; (2) a history of epilepsy or the presence of intracranial metal implants that would contraindicate the use of rTMS; (3) a history of bleeding disorders or any skin damage or infection in the abdominal area that would prevent the administration of AA; (4) a history of mental illness or severe cognitive or communication disorders that impair the result in poor compliance; (5) pregnant or lactating women.

Preliminary effect parameters for the primary outcome FMA-LE were extracted from the pilot trial of Kim et al. (18): the expected between-group mean difference was 3.6 points (rTMS vs. control), with a standard deviation of 4.5, corresponding to a standardized effect size Cohen’s d = 0.8. To achieve meaningful improvements with an alpha level of 0.05 and a beta of 80%, a power analysis revealed that a sample size of 30 participants per group was required. Taking into account a 20% attrition rate, we estimated a total sample size of 108 participants, with 36 assigned to each group.

### Randomization and blinding

After enrollment, a statistical software program was used to obtain a random number table. According to the order in which the participants entered the experiment and the corresponding groups of characters in the random number table, they were randomly divided into the control group, the AA group, and the combined group in a 1:1:1 ratio. The results of randomization were kept in randomly arranged opaque envelopes and were blinded for the assessors, data collectors, and statisticians.

All outcome assessors and data collectors were independent researchers who did not partake in group allocation or intervention delivery. Furthermore, all paper case report forms and electronic data sheets utilized unique participant identification numbers without any group-labeling information, and analysis was conducted by an independent statistician.

### Interventions

All patients who met the inclusion criteria received conventional medical treatment in conjunction with lower limb rehabilitation training. The control group was administered only conventional medical treatment alongside lower limb rehabilitation training. Patients assigned to the AA group received AA treatment in addition to the interventions provided to the control group. In the combined group, patients initially received AA treatment followed by rTMS therapy, in accordance with the control group’s treatment regimen.

### Conventional medical treatment and routine rehabilitation training

For individuals diagnosed with hemorrhagic stroke, active measures were implemented to regulate blood pressure and intracranial pressure, to monitor blood glucose levels, and to manage bleeding. In cases of ischemic stroke, strategies to enhance cerebral blood circulation were employed, which included pharmacological interventions such as intravenous thrombolysis, anticoagulation, volume expansion, and vasodilation.

The primary rehabilitative exercises encompassed passive joint movement, muscle strength training, practice of turning over and sitting up in bed, sitting balance exercises, bed-to-chair transfer, sitting-to-standing exercises, standing balance training, weight-shifting exercises, step training, walking practice, and activities of daily living ADL training. The treatment protocol involved 5 days of intervention per week, with each session lasting 30 min, continuing for a total of 8 weeks.

### AA treatment

Selection of acupuncture points: The main acupuncture points selected included Zhongwan (CV12), Xiawan (CV10), Qihai (CV6), and Guanyuan (CV4). Auxiliary points consisted of bilateral Tianshu (ST25) and Daheng (SP15) points. Points Huaroumen (ST24) and Wailing (ST26) on the affected side were also utilized.

Acupuncture procedure: The skin at the selected acupuncture points was subject to routine disinfection. A sterile disposable acupuncture needle measuring 0.35 mm x 40 mm (from Suzhou Hwato Medical Equipment Co., Ltd.) was then inserted into the subcutaneous tissue to a depth of 15-25 mm. After achieving qi through acupuncture, the needle was retained for a duration of 30 min without additional manipulation. This treatment was administered 5 days per week, over a total of 8 weeks.

Management of adverse events: In the event of complications during or after acupuncture treatment, such as sticking of needle, fainting, bent needles, or bleeding following needle removal, these occurrences were managed according to established protocols for handling acupuncture-related incidents, with relevant explanations provided to patients.

### rTMS treatment

The efficacy of rTMS is primarily influenced by stimulation frequency: low-frequency rTMS leads to a temporary decrease in cortical excitability, whereas high-frequency rTMS elevates it. In this trial, low-frequency rTMS stimulation was applied to the healthy hemisphere of the brain, leveraging the interhemispheric imbalance model. This method inhibits the excessive cortical excitability on the healthy side, thereby improving the control capacity of the motor centers and subsequently facilitating enhanced balance and motor function in patients.

Determining the resting motor threshold (RMT): Patients were instructed to sit or lie down comfortably while wearing a positioning cap to ensure relaxation. The stimulation coil was positioned over the lower limb motor cortex representation area of the healthy hemisphere (M1 region). Utilizing a single-pulse stimulation mode, the coil was engaged 5–10 times to ascertain the minimum stimulation intensity required to elicit action potentials in the target muscles, thereby establishing the individualized motor threshold.

Treatment protocol: Patients were maintained in the same posture while the coil was securely positioned over the lower limb motor function area of the healthy hemisphere. TMS parameters were set to a frequency of 1 Hz and an intensity of 90% of RMT, with each pulse sequence lasting 10 s followed by a 5-s interval, culminating in a total of 20 min and 1,000 pulses. Throughout the treatment, it was essential to ensure patient relaxation, head stability, and consistent coil positioning. This treatment regimen was conducted 5 days per week over 8 weeks.

Adverse event management: In cases where patients experienced adverse reactions such as nausea, dizziness, or vomiting that did not subside after rest, treatment would be promptly terminated, with symptomatic management provided as required.

### Outcomes

All patients in each group underwent assessments of the primary observation indicators before treatment and at 4 and 8 weeks after treatment. The rehabilitation assessment was conducted using an assessor-blind method, ensuring that the assessors were not involved in the grouping or treatment of the patients.

Motor function: The Fugl-Meyer Assessment for Lower Extremity (FMA-LE) was employed to evaluate various aspects of motor function, including reflex activities, joint stability, coordination, and speed. A lower score indicates a greater severity of impairment in limb motor function. This scale has demonstrated high reliability and validity and has been validated by numerous studies for its sensitivity to changes in lower limb motor function among stroke patients (19).

Balance function: The Berg Balance Scale (BBS) was utilized to assess balance through a range of tasks, from static to dynamic balance, including standing, sitting, turning, and single-leg support. A total score of less than 40 signifies a risk of falling. Research indicates that the BBS can accurately reflect patients’ balance abilities and effectively predict the risk of falls, providing crucial insights for rehabilitation treatment (20).

ADL: The Modified Barthel Index (MBI) consists of 10 items that assess activities such as eating, dressing, using the toilet, personal hygiene, transferring, walking, and stair climbing. A higher score reflects a greater capability in daily living activities. Studies have shown that the MBI is sensitive to changes in the daily living abilities of stroke patients and is closely linked to clinical outcomes (21).

Safety indicators: Throughout the treatment period, the occurrence of adverse reactions—including muscle soreness, local skin redness and swelling, dizziness, headaches, and nausea—was recorded, and the incidence of these reactions was calculated. All adverse events were classified and graded according to the Common Terminology Criteria for Adverse Events Version 5.0 (CTCAE v5.0). Mild adverse events were defined as transient minor symptoms that did not necessitate medical intervention and did not interfere with study procedures or the daily functioning of participants. A serious adverse event was defined as any incident leading to death, life-threatening status, hospital admission, permanent disability, or other clinically significant harm that required urgent medical treatment.

### Statistical analyses

Results were expressed as mean ± standard deviation (SD) or as a percentage (%). In this longitudinal clinical trial with repeated outcome measurements, Mixed-effects Model for Repeated Measures (MMRM) was adopted as the primary analytical approach to handle missing outcome data, which is the preferred method for modern clinical trials as recommended. Participants with incomplete follow-up assessments were not excluded from the analysis; all available repeated measurements from each subject were retained and incorporated into the model. All statistical outputs, *p*-values and effect sizes presented in tables and figures were exclusively generated from MMRM models. For sensitivity verification, multiple imputation (MI) with chained equations was conducted to impute missing outcome values, and re-analyses were performed using the imputed dataset. Categorical variables were assessed using the chi-square (χ^2^) test. The statistical analysis was performed using SPSS version 23.0. A *p*-value of less than 0.05 indicates a statistically significant difference.

## Results

### Baseline characteristic analysis

**Figure 1:**
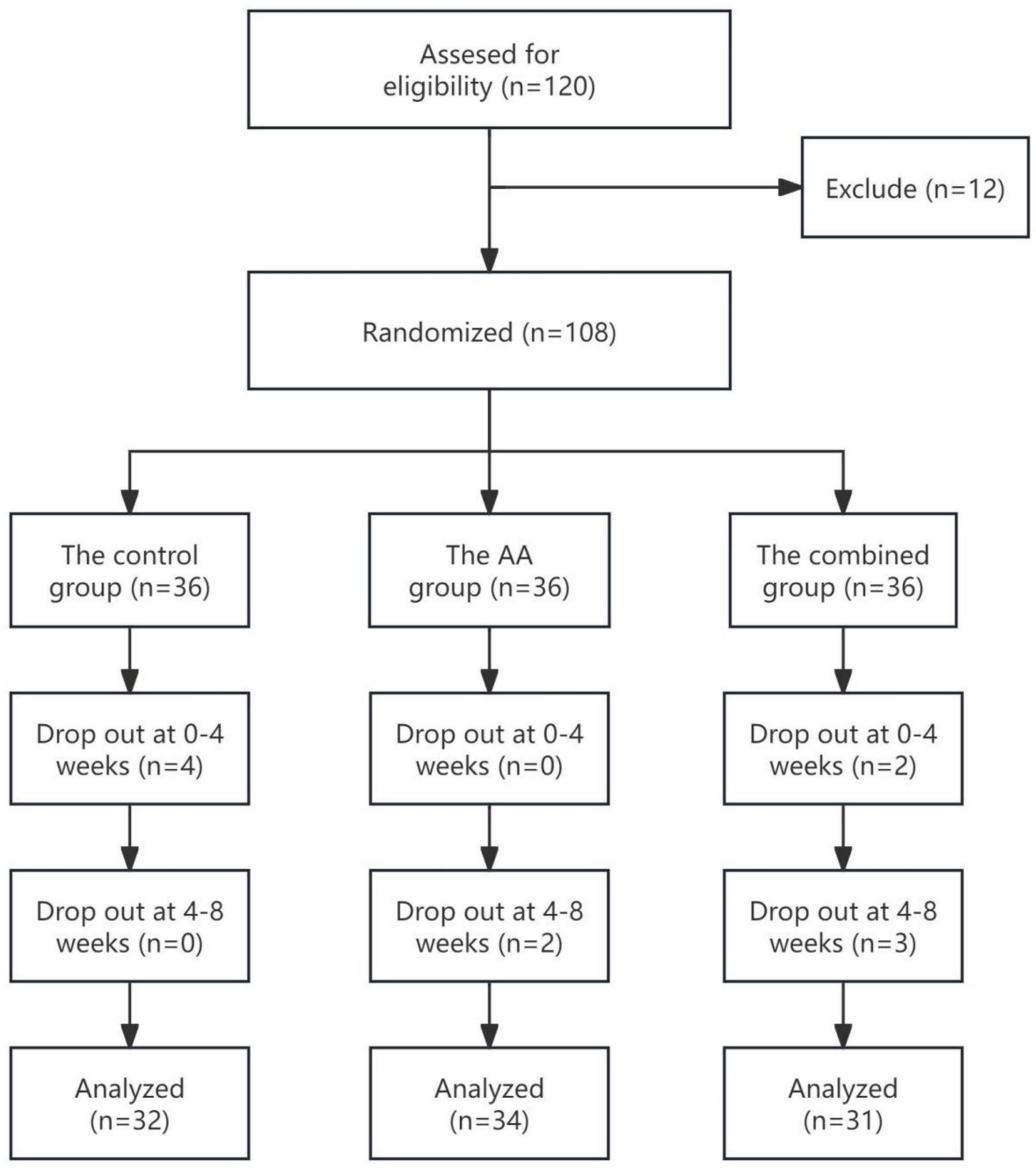
Trial flow chart.

A total of 108 patients were recruited for our study and randomly assigned to three groups: a control group, an AA group, and a combined treatment group, with each consisting of 36 patients. Throughout the study, several patients dropped out: 4 from the control group (0–4 weeks), 2 from the AA group (4–8 weeks), and 2 from the combined group (0–4 weeks), along with an additional 3 who dropped out (4–8 weeks) (Figure 1). The general information about the three groups is summarized in Table 1. Comparative analyses of baseline demographic and clinical evaluation data among the groups revealed no statistically significant differences (*p* > 0.05), thereby confirming the comparability of the groups at the outset of the study.

**Table 1:** Comparison of general data among the three groups of patients.

Variables	Control (*n* = 36)	AA (*n* = 36)	Combined (*n* = 36)	*p*
Male/female (n)	23/13	22/14	23/13	0.097
Age (y)	67.33 (14.61)	67.39 (13.93)	61.03 (14.08)	0.055
Hemorrhagic/ischemic stroke (n)	10/26	17/19	17/19	0.153
Left/right hemiplegia (n)	18/18	14/22	15/21	0.613
Course(d)	24.31 (16.08)	28.08 (21.06)	31.58 (16.46)	0.234
FMA-LE	13.03 (5.05)	13.28 (4.84)	13.19 (5.80)	0.979
BBS	16.47 (8.16)	14.58 (9.63)	15.97 (10.76)	0.688
MBI	29.08 (16.01)	27.03 (16.75)	30.56 (17.86)	0.674

AA, abdominal acupuncture; Combined, repetitive transcranial magnetic stimulation (rTMS) in combination with AA; FMA-LE, Fugl-Meyer Assessment of Lower Extremity; BBS, Berg Balance Scale; MBI, the Modified Barthel Index. Data are presented as mean ± (standard deviation).

### FMA-LE scores

**Figure 2:**
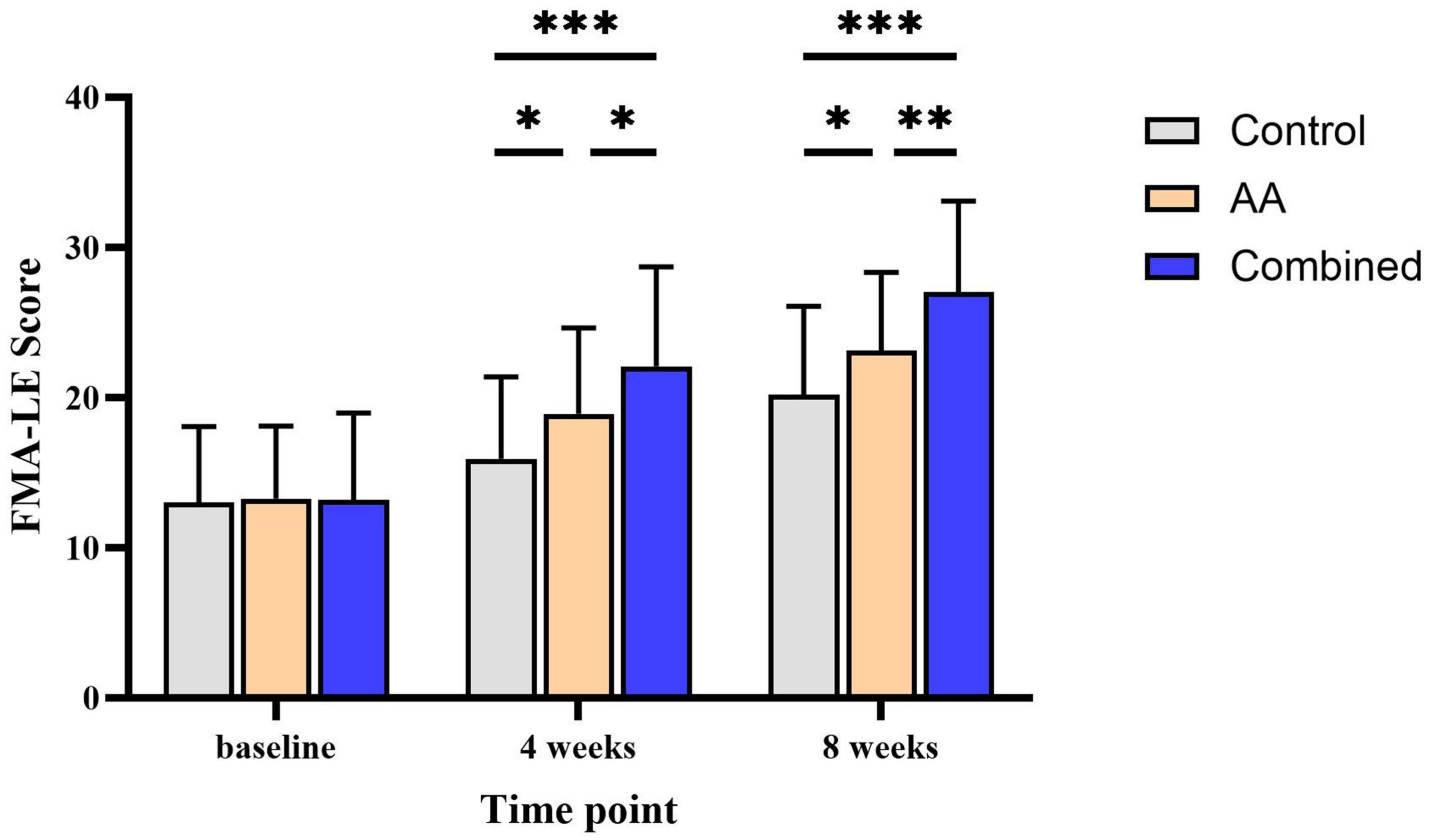
Comparison of FMA-LE scores among the three groups of patients. AA, abdominal acupuncture; Combined, repetitive transcranial magnetic stimulation (rTMS) in combination with AA; FMA-LE, Fugl-Meyer Assessment of Lower Extremity; *p < 0.05, **p < 0.01; ***p < 0.001.

The results demonstrated that the FMA-LE scores for all three groups showed a significant improvement compared to their baseline scores (*p* < 0.05). After 4 and 8 weeks of treatment, the FMA-LE scores of patients in the combined group were notably higher than those in both the control group and the AA group (Figure 2). At the 8-week evaluation, the average scores were (27.06 ± 6.05) for the combined group, (23.12 ± 5.24) for the abdominal acupuncture group, and (20.19 ± 5.90) for the control group (Table 2). The results established the hierarchy among the groups: the combined group exhibited a performance level that surpassed that of the AA group, which, in turn, outperformed the control group. The differences in scores between each pair of groups were statistically significant (*p* < 0.05).

**Table 2:** The number of patients and the FMA-LE scores at different time points.

Time points	Control		AA		Combined	
	*n*	Mean (SD)	*n*	Mean (SD)	*n*	Mean (SD)
Baseline	36	13.03 (5.05)	36	13.28 (4.84)	36	13.19 (5.80)
4 weeks	32	15.94 (5.45)	36	18.92 (5.73)	34	22.09 (6.64)
8 weeks	32	20.19 (5.90)	34	23.12 (5.24)	31	27.06 (6.05)

SD, standard deviation; AA, abdominal acupuncture; Combined, repetitive transcranial magnetic stimulation (rTMS) in combination with AA.

### BBS scores

**Figure 3:**
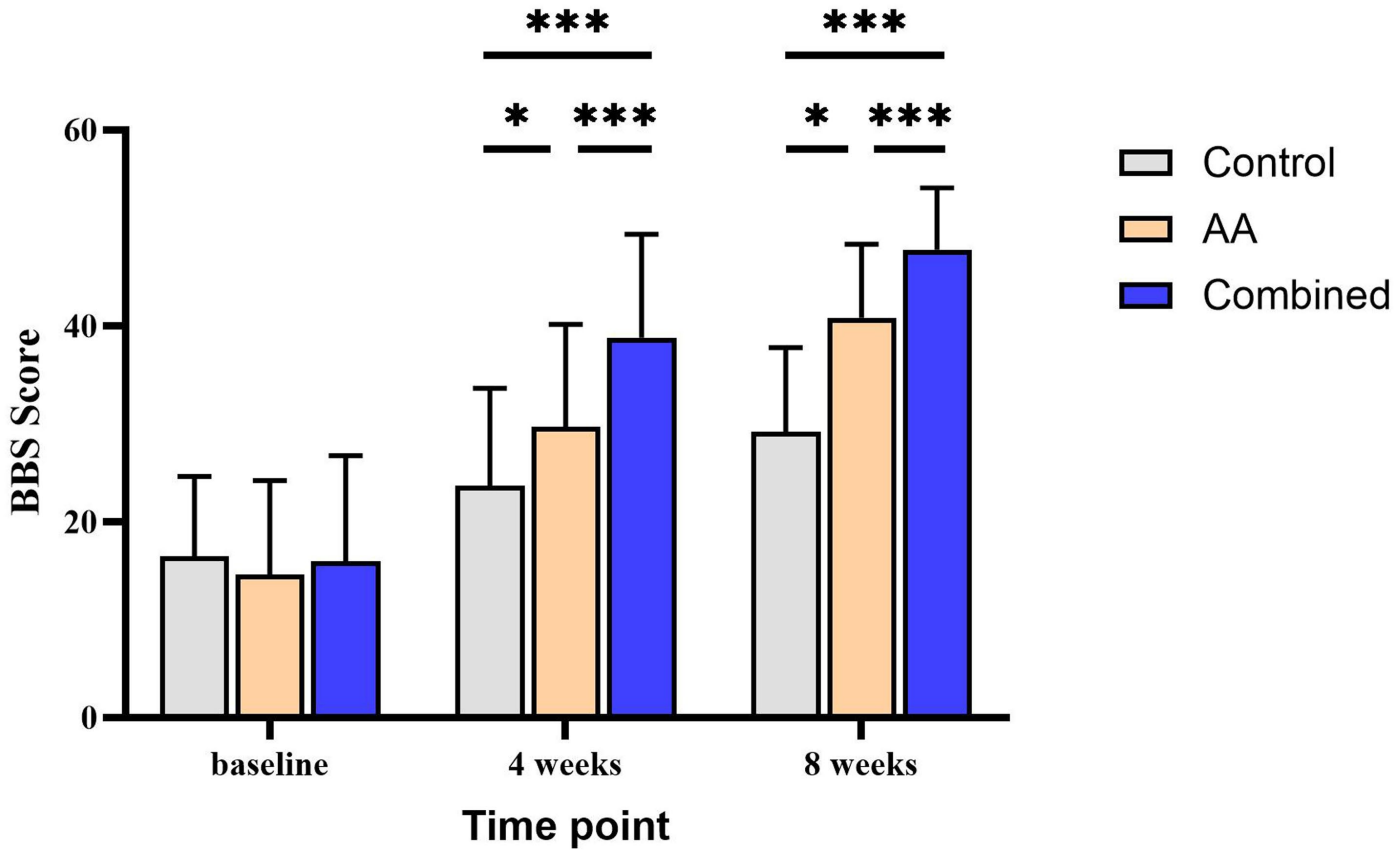
Comparison of BBS scores among the three groups of patients. AA: Abdominal acupuncture; Combined: Repetitive transcranial magnetic stimulation (rTMS) in combination with AA; BBS: Berg balance scale; *p < 0.05; **p < 0.01; ***p < 0.001.

As time progressed, the scores for each group exhibited a notable increasing trend, which was statistically significant (p < 0.05). After 4 and 8 weeks of treatment, the BBS scores for the combined group were significantly higher than those of the other two groups (Figure 3). The average scores at the eighth week were as follows: the combined group reached a mean of 47.77 ± 6.31 points, while the control group scored 29.16 ± 8.63 points, and the AA group achieved 40.82 ± 7.50 points (Table 3). Furthermore, the AA group outperformed the control group, with the difference being statistically significant (*p* < 0.05).

**Table 3:** The number of patients and the BBS scores at different time points.

Time points	Control		AA		Combined	
	*n*	Mean (SD)	*n*	Mean (SD)	*n*	Mean (SD)
baseline	36	16.47 (8.16)	36	14.58 (9.63)	36	15.97 (10.76)
4 weeks	32	23.69 (9.95)	36	29.72 (10.43)	34	38.79 (10.54)
8 weeks	32	29.16 (8.63)	34	40.82 (7.50)	31	47.77 (6.31)

SD, standard deviation; AA, abdominal acupuncture; Combined, repetitive transcranial magnetic stimulation (rTMS) in combination with AA.

### MBI scores

**Figure 4:**
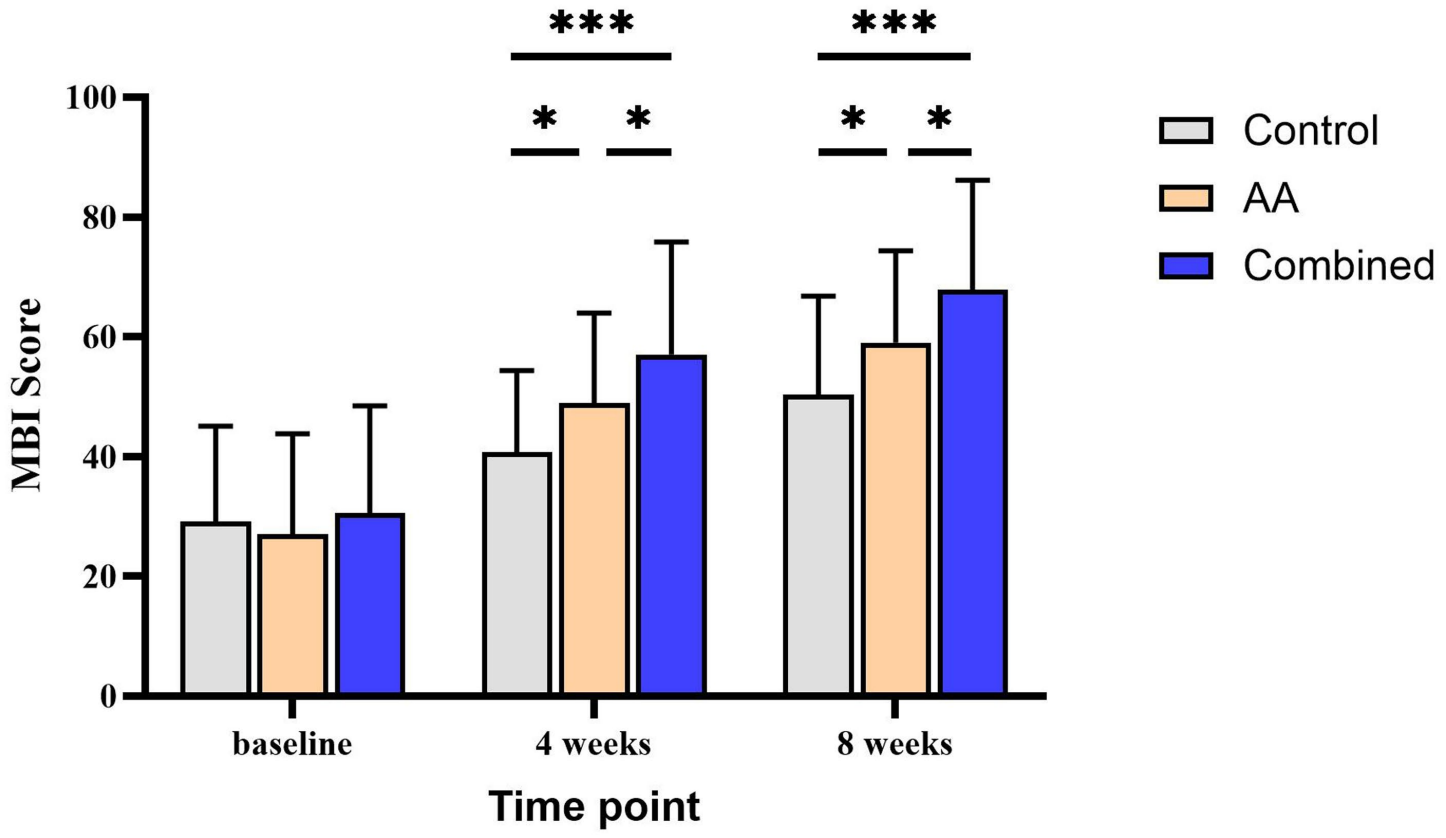
Comparison of MBI scores among the three groups of patients. AA: Abdominal acupuncture; Combined: Repetitive transcranial magnetic stimulation (rTMS) in combination with AA; MBI: The Modified Barthel Index; *p < 0.05, **p < 0.01; ***p < 0.001.

The indicators for all three patient groups demonstrated improvement at each time point following the intervention compared to their baseline measurements, with continued enhancement over time (p < 0.05). Statistical analysis revealed that, after 4 and 8 weeks of treatment, the MBI scores for the combined group significantly surpassed those of the other two groups (Figure 4). The average MBI score for the combined group was 67.87 ± 18.26 points after 8 weeks (Table 4). In contrast, the AA group recorded a score of 59.03 ± 15.34 points, which was notably higher than the control group’s score of 50.28 ± 16.51 points (*p* < 0.05).

**Table 4:** The number of patients and the MBI scores at different time points.

Time points	Control		AA		Combined	
	*n*	Mean (SD)	*n*	Mean (SD)	*n*	Mean (SD)
Baseline	36	29.08 (16.01)	36	27.03 (16.75)	36	30.56 (17.86)
4 weeks	32	40.69 (13.68)	36	48.92 (15.03)	34	57.03 (18.81)
8 weeks	32	50.28 (16.51)	34	59.03 (15.34)	31	67.87 (18.26)

SD, standard deviation; AA, abdominal acupuncture; Combined, repetitive transcranial magnetic stimulation (rTMS) in combination with AA.

### Between-group comparisons

Take the FMA-LE at 8 weeks for example, the mean difference between AA Group and Control Group was 2.93, with a 95% confidence interval of [0.19, 5.67] and *p* < 0.05, Cohen’s d = 0.53 (moderate effect); the mean difference between Combined Group and Control Group was 6.88, with a 95% confidence interval of [3.87, 9.89] and *p* < 0.001, Cohen’s d = 1.15 (large effect); there was statistically significant difference between Combined Group and AA Group, with a mean difference of 3.95, a 95% confidence interval of [1.15, 6.75], and *p* < 0.01, Cohen’s d = 0.70. The details of the other items can be found in Table 5.

**Table 5:** Between-group comparisons of FMA-LE, BBS, and MBI scores among the three groups.

Outcomes	Statistics	AA vs. Control	Combined vs. Control	Combined vs. AA
FMA-LE baseline	MD [95% CI]	0.25 [−2.08, 2.58]	0.17 [−2.39, 2.72]	−0.08 [−2.60, 2.43]
	Cohen’s d	0.05	0.03	−0.02
FMA-LE 4 weeks	MD [95% CI]	2.98 [0.26, 5.70]	6.15 [3.15, 9.15]	3.17 [0.22, 6.13]
	Cohen’s d	0.53	1.00	0.51
FMA-LE 8 weeks	MD [95% CI]	2.93 [0.19, 5.67]	6.88 [3.87, 9.89]	3.95 [1.15, 6.75]
	Cohen’s d	0.53	1.15	0.70
BBS baseline	MD [95% CI]	−1.89 [−6.09, 2.31]	−0.50 [−4.99, 3.99]	1.39 [−3.41, 6.19]
	Cohen’s d	−0.21	−0.05	0.14
BBS 4 weeks	MD [95% CI]	6.04 [1.08, 10.99]	15.11 [10.06, 20.15]	9.07 [4.07, 14.08]
	Cohen’s d	0.59	1.47	0.87
BBS 8 weeks	MD [95% CI]	11.67 [7.70, 15.64]	18.62 [14.80, 22.44]	6.95 [3.50, 10.40]
	Cohen’s d	1.45	2.46	1.00
MBI baseline	MD [95% CI]	−2.06 [−9.76, 5.65]	1.47 [−6.50, 9.45]	3.53 [−4.61, 11.67]
	Cohen’s d	−0.13	0.09	0.20
MBI 4 weeks	MD [95% CI]	8.23 [1.24, 15.22]	16.34 [8.21, 24.47]	8.11 [0.01, 16.21]
	Cohen’s d	0.57	0.99	0.48
MBI 8 weeks	MD [95% CI]	8.75 [0.92, 16.58]	17.59 [8.82, 26.36]	8.84 [0.51, 17.18]
	Cohen’s d	0.55	1.01	0.53

FMA-LE, Fugl-Meyer Assessment of Lower Extremity; BBS, Berg Balance Scale; MBI, the Modified Barthel Index; MD, mean difference; CI, confidence interval; AA, abdominal acupuncture; Combined, repetitive transcranial magnetic stimulation (rTMS) in combination with AA.

The minimum clinically important difference (MCID) represents the smallest score difference that patients and clinicians perceive as meaningful functional improvement. MCID thresholds for all three functional scales were sourced from stroke-specific validation literature to distinguish statistically significant group differences from patient-perceivable clinical benefits: 6-point MCID for FMA-LE, 5-point MCID for BBS, and 10-point MCID for MBI. For FMA-LE, AA versus control showed MD below 6 at week 4 (2.98) and week 8 (2.93), without clinically meaningful lower-limb improvements. The combined group reached clinical significance versus controls at both time points (6.15 and 6.88). No perceptible clinical difference existed between combined and AA groups (MD = 3.17, 3.95). For BBS, AA vs. control exceeded the 5 MCID at week 4 (slightly above 5) and 8 (markedly above 5). The combined group produced robust clinically meaningful balance gains versus controls and AA at both follow-ups (all MD > 5). For MBI, AA therapy failed to deliver clinically meaningful gains in daily self-care. Combined vs. Control MDs of week 4 and 8 surpassed 10, with substantial clinically relevant ADL improvements. However, the Combined and AA interventions showed no clinically distinguishable difference in ADL capacity.

### Adverse events

Throughout the treatment process, comprehensive records were kept regarding the adverse reactions experienced by patients across the three groups. During the treatment period, the following observations were made: In the control group, 2 patients reported mild muscle soreness following rehabilitation training, resulting in an adverse reaction rate of 5.56%. In the AA group, 2 patients exhibited local redness and swelling at the acupuncture site on the abdomen, while 1 patient experienced brief dizziness, leading to an adverse reaction rate of 8.33%. In the combined group, 2 patients experienced mild headaches, and 1 patient reported soreness at the acupuncture site, resulting in an identical adverse reaction rate of 8.33%. All adverse reactions in the three groups were relatively mild and resolved spontaneously without the need for special treatment. A Chi-square test analysis revealed no statistically significant differences in the adverse reaction rates among the groups (*p* > 0.05).

## Discussion

This randomized controlled trial examined the effects of rTMS in conjunction with AA on motor function, balance ability, and ADL in patients with post-stroke hemiplegia and lower limb dysfunction. The findings revealed that the combined treatment group achieved significantly higher scores across all three indicators compared to the AA group and the control group after treatment. According to the reanalysis utilizing the updated MCID thresholds, AA monotherapy yielded narrow therapeutic effects, resulting in mild yet clinically meaningful improvements in balance from week 4 onward. However, it did not achieve the clinical benchmarks for lower-limb motor function and ADL independence at either follow-up. In contrast, the combined intervention produced extensive and early clinically relevant improvements across all three assessments by week 4, along with larger functional gains observed by week 8. This suggests that the combination therapy is more effective in enhancing lower limb function in patients, displaying greater efficacy than either treatment used individually. Furthermore, there were no significant differences in the occurrence of adverse events among the three groups, and all reported adverse reactions were mild. This confirms the safety of the combined treatment plan and indicates that it is well-tolerated by patients.

The primary pathological mechanism behind lower limb dysfunction following a stroke, as understood in Western medicine, involves damage to neurons in the motor area of the cerebral cortex (22). This neuronal damage results in impaired control over limb movement and balance. Conversely, traditional Chinese medicine attributes this dysfunction to deficiencies in qi and blood within the internal organs and meridians, along with stasis in these meridians (23). Recovery of motor function in patients with hemiplegia post-stroke adheres to specific principles of neuroplasticity (24). Brain function remodeling serves as the physiological basis for rehabilitation, with the core mechanism involving activity-dependent synaptic reconnection and the reinforcement of neural network functions. This study concentrated on patients exhibiting lower limb dysfunction classified within Brunnstrom stages II to IV. These individuals typically present common movement patterns, challenges in establishing distinct movements, and abnormal fluctuations in muscle tone (25). To facilitate effective early intervention during this critical phase, a comprehensive, multidimensional collaborative strategy is essential. Standardized rehabilitation training can help suppress abnormal coordinated movements, foster separate movement patterns, regulate pathological muscle tone, and lay a solid foundation for the recovery of walking ability and balance (26).

In this study, the control group received conventional basic treatment in conjunction with rehabilitation training. This approach effectively enhanced lower limb muscle strength and improved joint range of motion through targeted exercises. However, it exhibited limited efficacy in regulating neural plasticity and facilitating the smooth circulation of meridians and qi. Conversely, the AA group received acupuncture at core acupoints in addition to the conventional treatment. The therapeutic effects observed in this group demonstrated statistically significant improvement of the balance function, thereby underscoring the unique efficacy of abdominal acupuncture in enhancing the stability of the abdominal and pelvic floor core muscles. The mechanisms underlying these therapeutic effects are consistent with the theory of biological holography, suggesting a holographic correspondence between specific acupoint areas on the abdomen and the lesion sites associated with functional disorders following a stroke. The acupuncture signals are transmitted to the central nervous system via this holographic reflection mechanism, targeting the activation of neural networks in the affected areas and facilitating functional remodeling (27). Within the AA system, the four acupoints—Zhongwan, Xiawan, Qihai, and Guanyuan—compose the ‘Guiding Qi to the Origin’ formula (28). Zhongwan and Xiawan, situated in the stomach region, bolster spleen function, enhance digestion, and nourish the body to generate qi and blood. Qihai nourishes primordial qi, while Guanyuan consolidates kidney energy and supports foundational health. Additionally, Tianshu, a large intestine acupoint, and Daheng, a key acupoint on the spleen meridian, are both located on the abdomen and correspond to the spleen and stomach. Moreover, given that the spleen is the origin of qi and blood generation, ensuring the smoothness of the abdominal meridians nourishes the blood vessels of the lower limbs, thereby improving conditions such as flaccidity, spasms, and movement control disorders (29). The acupoint Huaroumen regulates qi within the upper limb meridians, whereas Wailing specializes in addressing disorders of the lower abdomen and lower limbs. Collectively, these acupoints establish a multi-target and multi-level regulatory network, thereby enhancing the overall therapeutic impact.

rTMS is a non-invasive brain regulation technology that utilizes pulsed magnetic fields to induce biological electrical effects within the brain (15). This technique precisely targets the cortical areas associated with hemiplegia through sequential repetitive pulses. The therapeutic mechanism is based on regulating the excitability of the cerebral cortex, inhibiting abnormal neuronal discharges, and restoring the dynamic balance of neural conduction pathways (30). As a result, it helps to restore the central nervous system’s ability to control motor nerves, effectively alleviating the spasticity in the lower limbs of stroke patients with hemiplegia. Low-frequency stimulation serves to inhibit excessive excitation in the healthy hemisphere while facilitating the functional reorganization of the damaged neural network. Concurrently, rTMS enhances the excitability of the affected cortex, which helps regulate peripheral effectors (31). This process inhibits the overactivity of spastic muscle groups and improves both muscle strength and the control accuracy of the quadriceps femoris. Moreover, rTMS can multi-dimensionally regulate the metabolic environment within the brain, providing a biological basis for neural plasticity (32). Ultimately, this technology establishes the groundwork for reconstructing the coordinated contraction patterns of muscles during the walking cycle, leading to significant enhancements in motor function and walking ability.

The central-peripheral dual regulation framework has been verified in upper limb rehabilitation research, and our work extends this mature theoretical model to the field of lower limb functional recovery, which constitutes the core novelty of the present trial. Given the potential complementarity between the ‘local neural regulation’ of rTMS and the ‘overall qi-blood regulation’ of AA, combining the two may enhance rehabilitation outcomes through a dual regulatory mechanism that operates centrally and peripherally. rTMS targets low-frequency inhibition of neural plasticity in the healthy hemisphere, helping to restore balance between the two hemispheres. Simultaneously, AA improves the stability of the zang-fu organs, enhancing local circulation and proprioceptive input from the core muscles. Together, these approaches synergistically promote the remodeling of the ‘central-peripheral-central’ neural circuit. This synergy allows for the gains in muscle strength, spasticity control, and motor coordination, with simultaneous optimization of both static balance maintenance and dynamic posture adjustment abilities in patients. As motor control and balance function improve in tandem, patients experience significant advancements in ADL. This integrated approach, which combines traditional Chinese medicine and Western medical principles, aligns with modern rehabilitation medicine’s focus on multi-system integration treatment, offering an innovative solution for the precise rehabilitation of motor disorders post-stroke.

This study presents several limitations. Firstly, the effect size estimate was derived from a small pilot trial, which may yield unstable preliminary effect estimates. And the follow-up period was relatively short, which may not adequately capture the long-term efficacy. Several participants withdrew from the present trial, which may introduce potential attrition bias. Future larger-sample trials with stricter follow-up management are required to validate our findings. Additionally, heterogeneity of stroke etiology and lack of sham control were important limitations of the present study. Due to constraints in the research environment, the specific mechanisms underlying the combined treatment were not explored in depth. In particular, the neurophysiological and molecular biological mechanisms warrant further investigation. Moreover, the indicators used in this study rely on qualitative evaluations based on scales, which can introduce subjective biases. Future research should incorporate more objective and direct assessment methods, such as surface electromyography and functional magnetic resonance imaging. Finally, given that rTMS and AA are specialized interventions, implementing a double-blind approach is particularly challenging for both participants and therapists in this study.

## Conclusion

The integration of rTMS and AA proves to be an effective treatment for lower limb dysfunction in patients experiencing post-stroke hemiplegia. This combined approach notably enhances motor function and balance in the lower limbs, as well as improves ADL. Its positive synergistic effects surpass those of conventional rehabilitation therapies or AA alone. Additionally, this combination is both safe and reliable, presenting a promising new strategy for the rehabilitation of lower limb dysfunction following a stroke.

## Data Availability

The raw data supporting the conclusions of this article will be made available by the authors, without undue reservation.
